# Impact of clinical pharmacist-led medication management and education on tacrolimus therapeutic control

**DOI:** 10.3389/fmed.2026.1851388

**Published:** 2026-06-09

**Authors:** Nur Ozturk, Büşra Nur Çattık, Barış Malbora, Ayşe Burcu Akıncı, Başak Adaklı Aksoy, Dilek Ece, Tunç Fişgin, Müge Gündoğdu, Melek Erdem, Safiye Suna Çelen, Nilay Aksoy

**Affiliations:** 1 Graduate School of Health Sciences, Clinical Pharmacy PhD Program, Istanbul Medipol University, Istanbul, Türkiye; 2 Department of Clinical Pharmacy, School of Pharmacy, Istanbul Medipol University, Istanbul, Türkiye; 3 Pediatric Hematology and Oncology, Memorial Bahçelievler Hospital, Istanbul, Türkiye; 4 Pediatric Stem Cell Transplantation Unit, Medical Park Pendik Hospital, Istanbul, Türkiye; 5 Pediatric Hematology and Oncology, Medical Park Gaziosmanpaşa Hospital, Istanbul, Türkiye; 6 Pediatric Hematology and Oncology, Koc University Hospital, Istanbul, Türkiye; 7 Pediatric Hematology and Oncology, Medical Park Göztepe Hospital, Istanbul, Türkiye; 8 Department of Clinical Pharmacy, Faculty of Pharmacy, Altinbaş University, Istanbul, Türkiye

**Keywords:** bone marrow transplantation, clinical pharmacist, pediatric, tacrolimus, therapeutic drug monitoring

## Abstract

**Introduction:**

The management of Tacrolimus in pediatric bone marrow transplantation (BMT) presents significant challenges owing to its narrow therapeutic index and considerable pharmacokinetic variability. This study assesses the effects of clinical pharmacist interventions, particularly the resolution of drug-related problems (DRPs) and caregiver and nursing staff education, on stabilizing tacrolimus blood concentrations.

**Methods:**

This prospective interventional study was conducted across five hospitals in Istanbul from June 2024 to January 2026. The study comprised 65 pediatric patients, their caregivers, and 50 nurses. Clinical pharmacists identified Drug-Related Problems (DRPs) utilizing the PCNE V9.00 system and delivered both verbal and written educational interventions. Knowledge assessments and tacrolimus levels were evaluated at baseline, post-education, and at the 3-month follow-up.

**Results:**

Out of the 35 pharmacist interventions conducted, 77.1% were successfully resolved. The proportion of patients within the therapeutic range (5–20 ng/ml) increased from 52.3% at baseline to 84.4% following the educational intervention and remained at 82.5% after 3 months (*p* < 0.001). Both caregivers and nurses demonstrated substantial and enduring improvements in their pharmacological knowledge and rational medication-use behaviors (*p* < 0.001). Notably, elevated knowledge levels and the presence of “unresolved DRPs” (*p* < 0.05) were identified as critical factors affecting tacrolimus stability, thereby adding an essential layer of control alongside traditional markers such as C-reactive protein (CRP) and renal function.

**Discussion:**

Clinical pharmacist-led interventions substantially improve the management of tacrolimus therapy in pediatric bone marrow transplantation. Systematic education and proactive resolution of drug-related problems are essential for optimizing medication levels and safeguarding patient safety by addressing critical behavioral and systemic factors in post-transplant management.

## 1 Introduction

Solid organ and stem cell transplantation remain life-saving interventions for pediatric patients with end-stage organ disease. Tacrolimus (Tac), one of the most important calcineurin inhibitors (CNIs), is commonly administered to pediatric patients following solid organ transplantation (SOT) and hematopoietic stem cell transplantation (HSCT) as part of their immunosuppressive regimen to prevent allograft rejection (1). Despite its widespread use and proven efficacy, the clinical management of tacrolimus is notoriously challenging. The drug is characterized by a remarkably narrow therapeutic index and extensive intra- and inter-patient pharmacokinetic variability (2). To balance the prevention of immune rejection against the risk of severe adverse effects, stringent therapeutic drug monitoring (TDM) is required to maintain tacrolimus trough concentrations within target ranges, typically 5–20 ng/ml (3).

The consequences of deviating from this therapeutic window are severe. Subtherapeutic tacrolimus exposure dramatically increases the risk of acute cellular rejection, the development of *de novo* donor-specific antibodies, and ultimately, graft loss (4). Conversely, supratherapeutic accumulation precipitates a cascade of toxicities, including profound nephrotoxicity, neurotoxicity, new-onset diabetes after transplantation, and metabolic derangements (5). In pediatric populations, maintaining these stable blood concentrations is uniquely complicated by age-related metabolic differences, a higher rate of hepatic drug clearance, and continuous physiological changes associated with growth and development (1, 6).

Beyond biological variability, a primary driver of erratic tacrolimus exposure is the occurrence of medication-related errors and Drug-Related Problems (DRPs) (7). In the outpatient setting, pediatric transplant success is entirely reliant on the caregiver's health literacy and adherence (8). Caregivers are tasked with managing highly complex, lifelong medication regimens. However, they frequently lack crucial baseline knowledge regarding the necessity of strict 12-h dosing intervals, appropriate missed-dose management, and the avoidance of severe food-drug interactions (9). For example, because tacrolimus is extensively metabolized by intestinal and hepatic cytochrome P450 enzymes (specifically CYP3A4 and CYP3A5), the ingestion of CYP3A4 inhibitors such as grapefruit can lead to sudden, toxic spikes in systemic drug exposure (10).

Misunderstandings in these areas directly fuel high intra-patient variability, which is recognized as a leading predictor of poor long-term graft outcomes (11). Furthermore, these medication-related risks are not limited to outpatient care; systemic vulnerabilities also extend to the inpatient environment, where nursing staff responsible for medication administration on hospital wards face challenges in navigating complex pharmacological regimens (12).

A lack of standardized knowledge regarding optimal dosing timing and critical interactions, such as antibiotic-mineral chelation or proton-pump inhibitor absorption issues, can inadvertently destabilize a patient's pharmacokinetic profile during critical hospital admissions (13–15).

To mitigate these multifaceted risks, multidisciplinary models integrating clinical pharmacists are vital in hematopoietic stem cell transplantation (HSCT) (16). Acting as “pharmacy navigators” across all transplant phases, pharmacists enhance medication safety and adherence through medication reconciliation, toxicity monitoring, and targeted education (17). Furthermore, their proactive resolution of pharmacotherapy-related problems reduces medication errors, maintains target immunosuppressive levels, and ultimately improves clinical outcomes and patient quality of life (16, 17). Consistent with these broader benefits, evidence specific to pediatric cohorts further validates the necessity of this multidisciplinary approach. For instance, research conducted by Ozdemir in 2020 at a Turkish pediatric bone marrow transplantation unit demonstrated that active integration of clinical pharmacists significantly enhances both the detection and management of drug-related problems, particularly in pediatric HSCT settings (18). Despite these benefits, there remains a critical need to evaluate how such interventions directly influence high-risk medication management, specifically narrow therapeutic index drugs like tacrolimus, and the role of caregiver education in preventing post-transplant complications. Therefore, the present study was designed to assess the impact of clinical pharmacist interventions, specifically the resolution of drug-related problems and the education of caregivers, on tacrolimus blood concentrations, among pediatric patients undergoing bone marrow transplantation.

## 2 Materials and methods

### 2.1 Data collection and study timeline

The study focused on identifying pharmaceutical care needs and drug-related problems, optimizing tacrolimus blood concentrations, and assessing the effectiveness of pharmacist-led educational interventions for patients' caregivers, such as nursing staff or family members. This multicenter study was conducted over 18 months, from June 1, 2024, to January 1, 2026, at five hospitals in Istanbul, specifically within their Pediatric Hematology, Oncology, and Bone Marrow Transplantation Day Care Units.

An *a priori* power analysis was conducted using G^*^Power 3.1 to determine the minimum sample size required to ensure adequate statistical power for the chi-square test and to reliably evaluate the pre-defined hypotheses (19). The analysis indicated a necessary minimum sample size of *n* = 44. To account for potential attrition and loss to follow-up over the course of the study, the final target sample size was adjusted to *n* = 65.

This study was designed as a prospective clinical trial involving 65 pediatric patients in hematology and oncology, aiming to assess the comprehensive effects of structured clinical pharmacist interventions on tacrolimus blood levels and related clinical outcomes following bone marrow transplantation. To objectively evaluate the efficacy of these pharmacist-led interventions, a retrospective control group comprising 30 pediatric patients who received standard medical care without additional intervention or education was included. Additionally, to ascertain the direct impact of the intervention within the same subjects, the rates of achieving therapeutic tacrolimus levels during the 3 months preceding the intervention were calculated and compared with post-intervention data for a cohort of 30 patients; these findings were subsequently analyzed in conjunction with the overall results of the entire study population. The primary objectives of this research included the proactive identification of drug-related issues during pharmacotherapy, continuous therapeutic drug monitoring of tacrolimus in alignment with its narrow therapeutic index, objective evaluation of the effectiveness of structured pharmacist-led educational initiatives provided to parents, and the delivery of targeted in-service training by the clinical pharmacist based on baseline assessments of drug interaction awareness among the active multidisciplinary healthcare team.

The medical records of all patients admitted to the hematology department for hematopoietic stem cell transplantation (HSCT) were prospectively reviewed to evaluate the pharmaceutical care requirements of this population. To be included in the study, patients had to be between 0 and 18 years of age, have undergone a bone marrow transplant, reside in the same household as their informal caregivers (parents), and be willing to participate. Conversely, patients were excluded if they had a history of multiple organ transplants, possessed incomplete demographic or laboratory data, or if their informal caregivers desired to withdraw them from the study at any time. Data collection was performed using a combination of electronic medical records, laboratory diagnostic reports, structured questionnaires administered to informal caregivers (parents) and healthcare providers (nurses), and the PCNE framework to identify drug-related problems. Specifically, the Pharmaceutical Care Network Europe (PCNE) classification system, version 9.00 (20), was utilized as the standardized tool to systematically categorize and document these issues. This validated framework enabled the clinical pharmacist to accurately record the exact nature of each problem, its underlying causes, the proposed targeted interventions, and the final resolution status.

### 2.2 Education materials

Both verbal and written education were provided to the caregivers, defined in this study as the informal caregivers and nursing staff. These educational sessions aimed to improve knowledge of the correct use of tacrolimus, medication adherence, food–drug interactions, management of missed doses, and prevention of medication errors. The written materials were provided as a booklet, developed in accordance with the Patient Education Materials Assessment Tool for Printable Materials (PEMAT-P) guidelines (21). To ensure high quality, the initial draft of the booklet underwent a rigorous validation process. First, it was evaluated by two independent academics for linguistic and pedagogical validation to ensure the medical terminology was appropriate for the health literacy level of the target audience. Subsequently, the revised material was submitted to an expert panel comprising the thesis advisor and specialist physicians actively working in the Pediatric Hematology, Oncology, and Bone Marrow Transplantation Unit, to confirm its medical accuracy, current scientific relevance, and clinical clarity. Following this structured feedback and final expert approval, the educational booklet was finalized and standardized for use in the study.

### 2.3 Intervention protocol and assessment tools

#### 2.3.1 Educational interventions were tailored to participant roles

- Informal caregivers, “parents” received individualized counseling on administration techniques, side effect monitoring, and adherence strategies.
- Health-care providers, “nurses” participated in workshops covering pharmacokinetic principles, food-drug interactions, and therapeutic monitoring protocols.
- Physicians engaged in case-based discussions regarding dose optimization and DRP management.

Tacrolimus was analyzed as a therapeutic-control outcome rather than as a mean concentration. All tacrolimus results were classified as below therapeutic range (< 5 ng/ml), within therapeutic range (5–20 ng/ml), or above therapeutic range (>20 ng/ml).

Tacrolimus trough concentrations, alongside structured questionnaires assessing informal caregivers' knowledge of therapy, rational drug-use behaviors, and nurses' knowledge of therapy and food–drug interactions, were evaluated simultaneously at three time points: directly before the pharmacist-led education, at the first test following the education, and 3 months post-education.

The knowledge and attitude of the study group were assessed by using four different questionnaires:

1. Patient Tacrolimus Knowledge Scale (11 items; scored as true/wrong/don't know): evaluates informal caregivers' understanding of tacrolimus indications, administration protocols, and adverse effects. The questions were prepared based on patient drug information extracted from UpToDate (22).
2. Rational Drug Use Questionnaire (40 items; 5-point Likert Scale) (23): assesses informal caregivers' daily medication management, behavioral adherence, and home administration safety. Approval to use the questionnaire has been obtained from the responding author.
3. Nurse Tacrolimus Knowledge Scale (11 items; scored as true/wrong/don't know; Sup 1): Measures inpatient nursing staff's clinical competency regarding tacrolimus administration and missed-dose protocols (22).
4. Nurse Food-Drug Interaction Knowledge Scale (25 items; scored as correct/incorrect/don't know) (24): evaluates nurses' understanding of how specific dietary components alter medication pharmacokinetics. An approval letter from the scale's author has been received.

### 2.4 Statistical analysis

Statistical analyses were conducted utilizing IBM SPSS version 29.0. Independent t-tests, Mann–Whitney *U*-tests, and Chi-square tests were employed for baseline comparisons. Temporal changes were analyzed through Repeated-Measures ANOVA and McNemar's tests (45). Generalized Estimating Equations (GEE) and multivariate linear regression models were used to identify predictors of achieving target tacrolimus concentrations. Statistical significance was established at *p* < 0.05.

### 2.5 Ethical considerations

Before the initiation of the study, ethical approval was obtained from Istanbul Medipol University Ethics Committee (no: 1026-07/12/2023), and all study procedures were conducted in accordance with the principles outlined in the declaration of Helsinki.

## 3 Results

### 3.1 Study population characteristics

The mean age of the prospective cohort was 9.08 ± 3.2 years, and 58.5% were male. Baseline tacrolimus trough levels averaged 7.37 ± 2.3 ng/ml in the study group and 7.29 ± 2.5 ng/ml in the control group. No statistically significant differences in age, weight, gender, or baseline tacrolimus levels were observed between the prospective and retrospective cohorts (*p* > 0.05).

The mean caregiver age was 38.2 ± 7.8 years. Most questionnaires were completed by mothers (54, 83.1%). Most families reported balanced socioeconomic status, with 78.5% indicating that income equaled household expenses, while 18.5% reported income below expenses. Regarding the informal caregivers' education level, 36.7% completed high school, 28.3% attended vocational school, 25.0% attended university, and 10.0% completed primary education.

A total of 50 healthcare providers (nurses) were included in the study. The mean age of the nurses was 25.6 ± 4.5 years, with 3.5 ± 4.2 years of experience. Regarding gender distribution, 20 (40%) were male, and 30 (60%) were female. Additionally, the primary working departments of 36 (72%) nurses were pediatric hematology, while the remaining 14 (28%) worked in pediatric oncology. Forty four percent of the nurses had more than 3 years of experience, and 56% reported that their income was equal to household expenses (Table 1).

**Table 1:** The demographic characteristics of the study group.

Pediatric patients	
Age (years), mean ± SD	9.08 ± 3.2	
Weight (kg), mean ± SD	28.10 ± 9.3	
Characteristic	Sub-domain	*n* (%)
Patient gender	Male	38 (58.5%)
	Female	27 (41.5%)
Child education	Not attending school	37 (56.9%)
	Primary school	14 (21.5%)
	High school	7 (10.8%)
	Middle school	7 (10.8%)
Informal caregivers		
Age (years), mean ± SD	38.2 ± 7.8 years	
Characteristic	Sub-domain	*n* (%)
Survey respondent	Mother	54 (83.1%)
	Father	11 (16.9%)
Health insurance	Yes	44 (67.7%)
	No	21 (32.3%)
Socioeconomic status	Income equals expenses	51 (78.5%)
	Income below expenses	12 (18.5%)
	Income above expenses	2 (3.1%)
Are the parents relative	Yes	9 (13, 8%)
	No	56 (86, 2%)
Education	High school	22 (36.7%)
	Vocational school	17 (28.3%)
	University	15 (25.0%)
	Primary school	6 (10.0%)
	Other	5 (8.3%)
Healthcare-provider (nurses)		
Mean nurse age, years ± SD	25.6 ± 4.5	
Mean nurse experience, years ± SD	3.5 ± 4.2	
Characteristic	Sub-domain	*n* (%)
Gender	Male	20 (40%)
	Female	30 (60%)
Marital status	Single	33 (66%)

### 3.2 Drug-related problems identified using the PCNE V9.00 classification

A total of 35 pharmacist recommendations were made to address identified DRPs. No recommendation was required for 39 patients (60.0%). One recommendation was provided for 17 patients (26.1%), while two recommendations were required for nine patients (13.9%). Among them, there were 14 males (53.8%) and 12 females (46.15%). Among the 35 recommendations, 27 (77.1%) were accepted and implemented either fully or partially. Pharmacist interventions were implemented at multiple levels, including prescriber communication, counseling of patients' informal caregivers, and direct medication adjustments. Most interventions involved discussion with prescribers (25.7%) or direct communication for awareness (17.1%). At the patient level, counseling and caregiver engagement accounted for a substantial proportion of interventions. Among the 27 accepted interventions, 18 (66.7%) resulted in complete resolution of the DRP, four (14.8%) were partially resolved, and five (18.5%) remained unresolved.

To quantify the impact of clinical pharmacist-led resolution of drug-related problems (DRPs), absolute risk reduction (ARR) and number needed to treat (NNT) were calculated. Patients were categorized into three groups based on DRP outcomes: no DRPs (*n* = 39), resolved DRPs (fully or partially resolved; *n* = 22), and unresolved DRPs (*n* = 5). The proportion of patients achieving in-range therapeutic target levels was 82.1% (*n* = 32) in the no DRPs group, 81.8% (*n* = 18) in the resolved DRPs group, and 40.0% (*n* = 2) in the unresolved DRPs group. Conversely, Out-of-therapeutic-range of Tacrolimus levels were observed in 17.9% (*n* = 7), 18.2% (*n* = 4), and 60.0% (*n* = 3) of these groups, respectively.

When comparing the resolved and unresolved DRP groups, the risk of out-of-therapeutic-tacrolimus range was 0.182 and 0.60, respectively. This comparison yielded an absolute risk reduction (ARR) of 0.418 and a calculated number needed to treat (NNT) of 2.4. All corresponding outcomes are presented in Table 2.

**Table 2:** Drug-related problems identified using the PCNE classification.

The problems	Primary domain	Cause	Number (*n*)	Percentage (%)
Problem		
Treatment effectiveness	Drug selection	Inappropriate drug according to guidelines/formulary	6	17.1
		No or incomplete drug treatment despite existing indication	7	20.0
		No indication for drug	5	14.28
Dose selection	Drug dose too low	3	8.57
Treatment safety	Adverse drug event	Dosage regimen too frequent	5	14.28
Other problems	Patient related	Inappropriate timing or dosing intervals	7	20.0
		Patient takes food that interacts	2	5, 71
Category		Status	Number (*n*)	Percentage (%)
Intervention				
Prescriber level		Prescriber informed only	6	17.1
		Intervention discussed with prescriber	9	25.7
		Intervention proposed to prescriber	5	14.3
Patient level		Patient (drug) counseling	4	11.4
		Spoken to family member/caregiver	5	14.3
Drug level		Dosage change	3	8.6
		Formulation change	3	8.6
Category		Status	Number (*n*)	Percentage (%)
Acceptance				
Intervention accepted		Intervention accepted and fully implemented	22	62, 85
		Intervention accepted, partially implemented	5	14, 28
Intervention not accepted		Intervention not accepted: not feasible accepted	5	14, 28
		Intervention not accepted: other reason (“changes in literature or previous fail of the suggested therapy”)	3	8, 57
DRP status		Total (*n*)	Tacrolimus in range *n* (%)	Tacrolimus out of range *n* (%)
DRP status				
No DRPs		39	32 (82.1%)	7 (17.9%)
Solved and partially solved		22 (18–4)	18 (81.8%)	4 (18.2%)
Unresolved DRPs		5	2 (40.0%)	3 (60.0%)
Comparison		Risk (out of range)	Number needed to treat (NNT)	Absolute risk reduction (ARR)
Absolute risk reduction and number needed to treat				
Resolved vs. unresolved DRPs		0.60 vs. 0.182	2.38	0.418

To illustrate the clinical impact of these actions, the following are representative examples of pharmacist interventions categorized by their PCNE V9.00 classification domains:

- Intervention for untreated indications (treatment effectiveness): the clinical pharmacist successfully initiated missing therapies, including adding trimethoprim/sulfamethoxazole for routine Pneumocystis Jirovecii Pneumonia (PJP) prophylaxis.
- Intervention for unnecessary drug therapy (treatment effectiveness): a clinical pharmacist successfully recommended tapering unindicated daily omeprazole in a post-transplant patient to safely reduce unnecessary polypharmacy and pill burden.
- Intervention for food-drug interactions (patient-related problems): the clinical pharmacist provided targeted dietary counseling on grapefruit consumption, which the family fully adopted.
- Intervention for inappropriate dosing intervals (patient-related problems): to resolve tacrolimus fluctuations, the clinical pharmacist counseled the informal caregivers on the absolute necessity of strict 12-h dosing intervals, successfully correcting the home administration schedule.
- Intervention for adverse drug event risks (treatment safety): the clinical pharmacist intercepted a safety risk: amlodipine was incorrectly ordered twice daily instead of the standard once-daily pediatric dose. In this case, the intervention was not accepted because the prescriber provided a clinical rationale for split-dosing based on the individual patient's rapid metabolism.

### 3.3 Informal caregivers' outcomes: tacrolimus knowledge scale and rational drug use questionnaire

The evaluation of parental comprehension of tacrolimus therapy across three distinct phases prior to intervention, immediately after intervention, and at a 3-month follow-up is succinctly outlined in Supplementary Table 1. Initially, parents demonstrated a high level of understanding regarding routine medication administration, such as adhering to the same daily intake time (90.8%) and every 12 h (90.8%). Conversely, their knowledge was comparatively limited concerning critical clinical scenarios, including the necessity for continued post-transplant medication use (18.5%), dose management following vomiting episodes (36.9%), and adherence to the 6-h dosing rule after missed doses (16.9%). At the 3-month mark, the majority of educational gains were sustained; parents maintained perfect accuracy (100%) in understanding the medication's primary objective to prevent organ rejection (A1) and the importance of administering the medication prior to meals (A9). A marginal decline was observed in more complex directives, such as the requirement for prolonged post-transplant therapy (reducing from 100% immediately post-intervention to 76.2%) and the 2-h rule following vomiting (decreasing from 95.3 to 82.5%). Nevertheless, the aggregate knowledge scores remained markedly higher than the baseline levels prior to the intervention (*p* < 0.05).

Concerning the rational drug use questionnaire, statistically significant enhancements were observed in parents' attitudes and behaviors after the educational intervention. Specifically, the “Strongly Agree” proportions for clinically critical items, including understanding the medication's purpose (B2), preparing suspensions (B5), recognizing side effects (B6), avoiding medications recommended by others (B10), and refraining from self-discontinuation upon experiencing side effects (B13), achieved 100% immediately post-intervention and were maintained at the 3-month follow-up (*p* < 0.001). Furthermore, compliance with shaking suspensions prior to administration (B17), which initially demonstrated one of the lowest baseline adherence levels, exhibited a highly significant increase in the “Strongly Agree” rate from 20.00 to 96.88% (*p* < 0.001). Statistically significant improvements were also observed in technical administrative procedures, including the disposal of expired medications (B26) and the use of designated measuring spoons (B24; *p* < 0.05). An analysis of negatively phrased items evaluating erroneous practices demonstrated a significant increase in the rates of “Strongly Disagree” responses for behaviors such as mixing medications (B16), opening capsules (B32), and administering drugs with milk (B36) or fruit juice (B40; *p* < 0.001). Additionally, parents exhibited markedly improved attitudes compared to baseline regarding the avoidance of leftover household medications (B30-B31) and the importance of consulting a physician in the case of adverse effects (B34-B35-B37-B38; Supplementary Table 2).

### 3.4 Nursing outcomes: medication management and food-drug interaction knowledge

The knowledge levels of 50 participating nurses regarding tacrolimus administration were assessed at three distinct time points: prior to the intervention, immediately following the intervention, and 3 months after the intervention. Analyses indicated statistically significant changes over time in nurses' responses to assertions regarding the primary purpose of the medication in preventing organ rejection (*p* < 0.001), the necessity for prolonged or lifelong use post-transplantation (*p* < 0.001), and protocols for dose management in the event of vomiting within the initial 2 h of administration (*p* < 0.001). Furthermore, significant alterations were observed in responses related to the importance of taking the medication at the same time every day (*p* = 0.014), the use of concurrent herbal supplements (*p* < 0.001), the immunosuppressive mechanism of action of the drug (*p* = 0.002), the recommendation for pre-prandial administration to address gastrointestinal issues (*p* < 0.001), the contraindication of grapefruit and grapefruit juice (*p* < 0.001), and the specific time-window guidelines for managing missed doses (*p* < 0.001). Conversely, no statistically significant changes were identified across the three time points concerning statements about the erroneous discontinuation of medication once the patient feels well (*p* = 0.132) and the necessity of maintaining strict 12-h intervals for twice-daily regimens (*p* = 0.132; Supplementary Table 3).

Nurses' knowledge of food-drug interactions was assessed at three time points: pre-intervention, immediately post-intervention, and 3 months post-intervention. Analyses showed highly significant improvements over time (*p* < 0.001) in responses to 24 specific statements covering a broad range of pharmacological principles. These included the general impact of fasting on drug efficacy, the need to administer antiulcer medications such as sucralfate on an empty stomach because of protein binding, and the effects of macronutrient-altered diets (energy-restricted, high-fat, high-carbohydrate, high-fiber, and low-protein) on the pharmacokinetics of drugs such as amphetamines, lipophilic agents, theophylline, propranolol, and digoxin. Furthermore, statistically significant changes were observed in knowledge of specific dietary interactions, including the effects of grapefruit juice on chemotherapeutics; caffeinated beverages on bisphosphonates; vitamin K-rich vegetables on warfarin; protein-rich foods on levodopa and methyldopa; and the chelation of antibiotics (tetracycline, ciprofloxacin) with essential minerals. Overall, differences across the three time points were statistically significant (*p* < 0.05) for all 25 evaluated statements. Comprehensive item-specific responses for the food-drug interaction knowledge scale are provided in (Supplementary Table 4).

### 3.5 Impact on tacrolimus blood concentrations

In the prospective group, the proportion of patients within the target tacrolimus therapeutic range (5–20 ng/ml) increased from 52.3% prior to intervention to 84.4% immediately following the intervention. It remained at 82.5% at the 3-month follow-up (*p* < 0.001). Sub-therapeutic levels decreased from 43.1% at baseline to 9.5% at 3 months (Table 3).

**Table 3:** Distribution and comparison of therapeutic target range attainment across time points in the control and education groups.

Timeline	Below range *n* (%)	Within range *n* (%)	Above range *n* (%)	*p*-value
Control group				
1 month before intervention (*n* = 30)	16 (53, 3)	13 (43, 3)	1 (3, 3)	0.002
1 month before intervention (*n* = 30)	9 (30, 0)	19 (63, 3)	2 (6, 7)	< 0.001
Immediately before intervention (*n* = 30)	9 (30, 0)	20 (66, 7)	1 (3, 3)	< 0.001
Education group				
Immediately before education (*n* = 65)	28 (43, 1)	34 (52, 3)	3 (4, 6)	< 0.001
First test post-intervention (*n* = 64)	9 (14, 1)	54 (84, 4)	1 (1, 6)	< 0.001
3 months post-intervention (*n* = 63)	6 (9, 5)	52 (82, 5)	5 (7, 9)	< 0.001
*p*-value (over time)	< 0.001	< 0.001	0.236	
Comparison	McNemar χ^*2*^			
Pre-education and post-education	12.45			< 0.001

*n*, number of participants.

The GEE model indicated that, following the educational intervention, patients exhibited a 5.41-fold increase in the likelihood of attaining the therapeutic range compared to baseline (95% CI: 2.59–11.30, *p* < 0.001). Moreover, the resolution of a DRP was associated with a 3.12-fold increase in the probability of reaching target levels (*p* = 0.003; Table 4).

**Table 4:** Adjusted generalized estimating equations (GEE) model for being in the tacrolimus therapeutic range.

Predictor (factor)	Adjusted OR (95% CI)	*p*-value
First laboratory test post-education compared to immediately pre-education (*n* = 65)	5.41 (2.59–11.30)	< 0.001
3 months post-education compared to 3 months pre-education (*n* = 30)	4.51 (1.94–10.48)	< 0.001
Patient age (for each one-year increase, *n* = 65)	1.12 (1.00–1.26)	0.053
Gender (female compared to male, *n* = 65)	2.14 (0.89–5.16)	0.090
Parents' age (for each one-year increase, *n* = 65)	1.01 (0.95–1.08)	0.732
Health insurance status (present compared to absent, *n* = 65)	1.24 (0.49–2.94)	0.693
Parents' tacrolimus knowledge score (high compared to low)	1.28 (1.10–1.49)	0.002
Caregiver's rational drug use score (high compared to low)	1.34 (1.15–1.56)	< 0.001
Nurses' tacrolimus knowledge score (high compared to low)	1.21 (1.05–1.40)	0.009
Nurses' food-drug interaction knowledge score (high compared to low)	1.26 (1.08–1.46)	0.003
Drug-related problem (DRP) status (absence compared to presence)	1.72 (1.02–2.94)	0.041
Pharmacist recommendation (accepted compared to not accepted)	2.75 (1.32–5.71)	0.006
DRP resolution status (resolved compared to unresolved)	3.12 (1.48–6.57)	0.003

The dependent variable is a binary variable (within range or outside range).

CI, confidence interval; OR, odds ratio.

### 3.6 Correlation and regression analysis

No significant correlations were found between tacrolimus trough concentrations and routine renal markers (eGFR, creatinine), hepatic enzymes (AST, ALT), electrolytes, or inflammatory markers (CRP, WBC; *p* > 0.05; Table 5).

**Table 5:** Correlation between tacrolimus trough concentrations and clinical parameters.

Category	Clinical parameter	Correlation coefficient (*r*)	*p*-value
Renal functions	Estimated glomerular filtration rate (eGFR)	−0.24	0.078
	Serum creatinine	0.21	0.096
Hematological parameters	Hemoglobin	−0.18	0.134
	Hematocrit	−0.16	0.158
	Albumin	−0.10	0.321
Liver function	Aspartate aminotransferase (AST)	0.19	0.112
	Alanine aminotransferase (ALT)	0.17	0.139
Electrolytes and metabolic	Magnesium	−0.22	0.084
	Potassium	0.18	0.121
	Glucose	0.09	0.356
Inflammatory markers	C-reactive protein (CRP)	0.23	0.082

**Figure 1:**
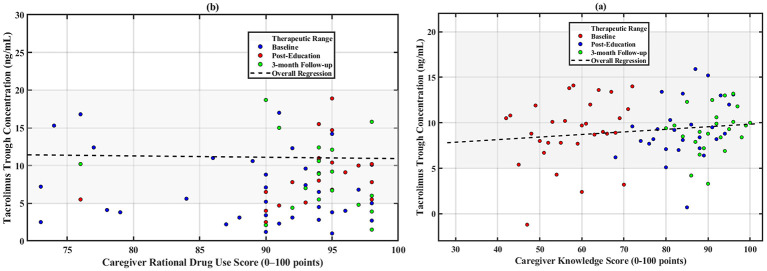
Regression plots of informal caregiver knowledge and rational drug use scores against tacrolimus levels and therapeutic control.

**Figure 2:**
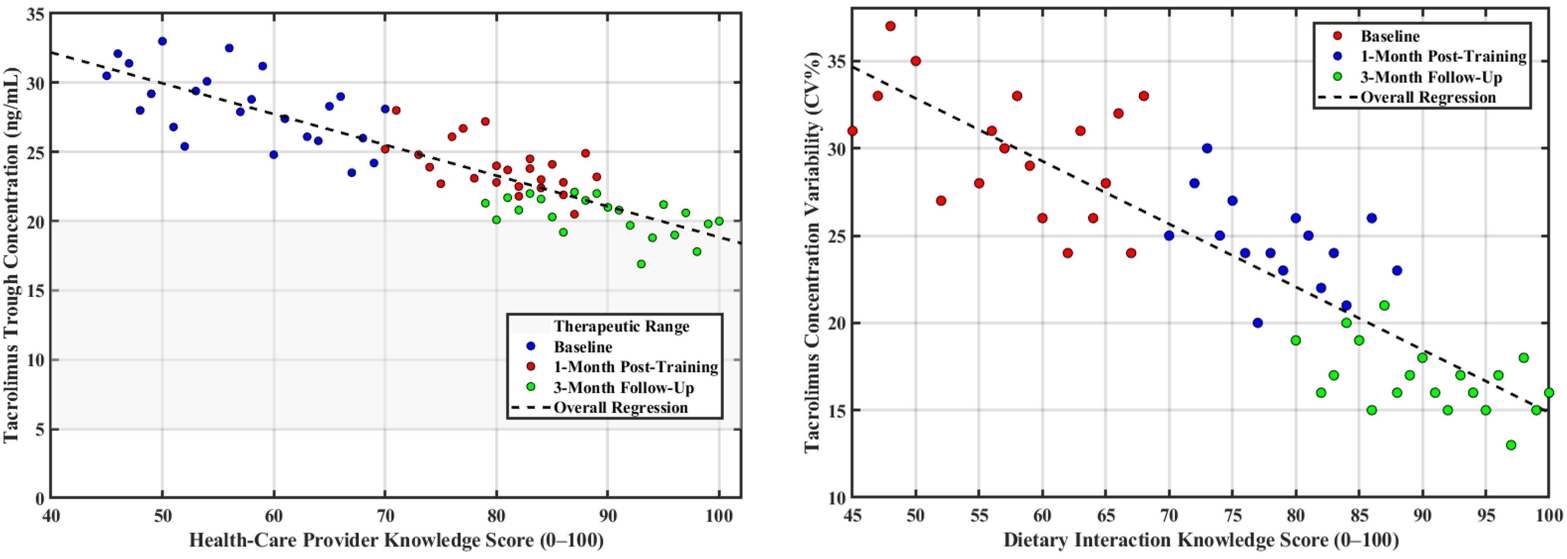
Regression plots of nurses' knowledge and food–drug interaction knowledge against tacrolimus therapeutic control.

The regression plot demonstrated that the informal caregivers' knowledge score was a significant positive predictor of tacrolimus levels (*B* = 0.34, SE = 0.12, *p* = 0.006). Similarly, rational drug use scores were statistically significant predictors of therapeutic control with tacrolimus (*B* = 0.29, SE = 0.11, *p* = 0.018; Figure 1). As shown in Figure 2, nurses' knowledge score was a significant predictor of tacrolimus therapeutic control (*B* = 0.31, SE = 0.13, *p* = 0.012). Regression analysis also revealed a statistically significant positive association between food–drug interaction knowledge and tacrolimus therapeutic control (*B* = 0.28, SE = 0.12, *p* = 0.021).

## 4 Discussion

Pediatric transplant recipients constitute a highly vulnerable demographic that necessitates lifelong, intricate immunosuppressive regimens to prevent graft rejection (25). The unique physiological and developmental changes characteristic of this age group, combined with the complexity of their treatments, increase their susceptibility to medication errors, non-adherence, and adverse drug reactions. Therefore, there exists an essential requirement for comprehensive, multidisciplinary pharmaceutical care specifically tailored to these patients (26). The initial and fundamental step in providing effective pharmaceutical care involves the systematic identification and evaluation of patient-specific needs and drug-related problems (DRPs) (27). By proactively identifying these issues, clinical pharmacists can develop targeted interventions to enhance medication safety and therapeutic efficacy (27).

The primary finding of this study is that a clinical pharmacist-led educational and medication management intervention significantly improves therapeutic-range control of tacrolimus by addressing drug-related problems (DRPs), enhancing knowledge, and accelerating therapeutic stabilization. The resolution of DRPs by clinical pharmacists effectively restores tacrolimus therapeutic control to levels comparable to those observed in patients without DRPs. Unresolved DRPs are associated with a markedly higher risk of out-of-therapeutic-range tacrolimus levels, with the number needed to treat (NNT) calculated as 2.38, indicating that approximately two to three pharmacist-led DRP resolutions are needed to benefit one additional patient by achieving therapeutic-range tacrolimus levels. These findings underscore the considerable clinical significance of clinical pharmacist intervention in optimizing tacrolimus therapy. Schuh and Massoglia demonstrated a 96% improvement in the proportion of patients attaining therapeutic tacrolimus levels following clinical pharmacist consultations post-bone marrow transplantation (28).

In our study, the therapeutic range of tacrolimus has increased significantly over the study period. Similarly, Massoglia et al. reported a 46% increase in the number of tacrolimus trough levels falling within the therapeutic range following pharmacist-led medication education and assessment (29). Another prospective evaluation of bone marrow transplant recipients emphasized that the inability to maintain immunosuppressive drugs within their optimal therapeutic range is a primary risk factor for graft complications, making pharmacist-led DRP resolution essential for patient safety (30). Literature evaluating transplant recipients consistently shows that unresolved DRPs, such as unrecognized drug-drug interactions, inappropriate dosing intervals, or non-adherence, are the primary drivers of erratic tacrolimus trough levels (31). Furthermore, studies demonstrate that when clinical pharmacists actively identify and resolve these issues using standardized classifications (like the PCNE tool), the proportion of patients achieving target therapeutic ranges increases significantly (32). These directly mirror our favorable outcomes in the resolved group. Our findings precisely mirror these concerns; interventions frequently addressed the need for additional therapy (20.0% of DRPs), such as initiating missing Pneumocystis Jirovecii Pneumonia (PCP) prophylaxis, and eliminating unnecessary drug therapy (17.1%), such as deprescribing unindicated proton pump inhibitors. A study conducted in the General Surgery Ward of an Academic Referral Hospital in Jordan reported the same DRPs, with the most common categories: “needs additional therapy” 46 (23.96%) and “unnecessary drug therapy” 45 (23.44%) (33). Our study found that unresolved DRPs were a strong independent predictor of increased tacrolimus variability compared with patients without DRPs, indicating a substantial impact of unresolved medication-related issues on the tacrolimus therapeutic range. In the same vein, the literature emphasizes that traditional, reactive dose adjustments often result in prolonged periods of subtherapeutic exposure. Conversely, proactive, pharmacist-led pharmacokinetic monitoring and immediate DRP resolution significantly reduce the time required to reach steady-state therapeutic levels (34).

In our study, before the educational intervention, informal caregivers' knowledge levels were variable, with a relatively high proportion of incorrect or uncertain responses across several items. Questions related to drug–food interactions, missed-dose management, and medication timing exhibited lower baseline accuracy. Post-intervention analysis showed substantial improvements in both knowledge and medication-use behaviors. Informal caregivers demonstrated better adherence to dosing schedules, improved understanding of drug interactions (especially grapefruit restriction), and more appropriate responses to missed doses and side effects. Because tacrolimus is heavily metabolized by the CYP3A4 pathway (which grapefruit strongly inhibits). Similarly, the literature emphasizes that intensive caregiver education on these specific dietary interactions is essential to avoid sudden, toxic spikes in systemic exposure (35). Research indicates that errors in pediatric medication administration in the home environment are common, with caregivers frequently making mistakes related to the frequency or duration of dosing, including missed doses (36). Furthermore, caregivers often struggle with complex medication schedules, and those with lower health literacy are at an even higher risk of making errors when administering medications to children (37).

Regarding the patient rational drug use survey, at baseline, several questionnaire items showed moderate levels of uncertainty or disagreement regarding appropriate medication practices. These included checking expiration dates, consulting health professionals before using medications, reading medication instructions carefully, and avoiding unnecessary medication use. For consistency, a study reported that caregivers exhibited moderate uncertainty about fundamental practices such as checking expiration dates, reading instructions, and avoiding unnecessary medications, consistent with recent research indicating that rational drug use literacy often remains critically low in community and caregiver populations (38). Another study on adherence barriers in pediatric kidney transplantation confirmed that minimizing adherence barriers and ensuring consistent home administration are key to maintaining stable drug exposure and preventing late acute rejection (39).

The food–drug interaction questionnaire was also analyzed separately in our study. All 25 items showed significant shifts in response distribution across time. The greatest improvements were seen in items involving grapefruit interactions, warfarin–vitamin K interactions, levodopa/protein interactions, antibiotic–mineral chelation, ACE inhibitor/potassium-rich foods, proton-pump inhibitor/iron absorption, and MAOI–tyramine interactions. A recent cross-sectional study evaluating healthcare professionals found that a significant proportion of nurses exhibit low-to-moderate baseline knowledge of complex Food Drug Interactions (FDI), highlighting a systemic vulnerability in inpatient care (40). Research shows that in-service training courses delivered by clinical pharmacists not only significantly increase nurses' knowledge but also directly reduce the rate of incorrect drug administration and clinically significant absorption interactions on hospital wards (41). A 2022 study evaluating kidney transplant recipients found a 46% increase in the proportion of tacrolimus trough levels within the therapeutic range following face-to-face pharmacist post-transplant consults (29). Similarly, a 2025 cohort study showed that patients managed with pharmacist interventions had significantly improved attainment of the target tacrolimus trough concentration compared to pre-intervention cohorts (42).

The linear regression analysis demonstrated that educational and pharmaceutical interventions, rather than routine clinical parameters, serve as the primary predictors of optimal tacrolimus trough concentrations. Specifically, enhanced caregiver and nursing knowledge regarding tacrolimus administration emerged as a statistically significant predictor of achieving therapeutic levels. These findings align with existing literature, which underscores the role of targeted education in reducing caregiver burden and improving the overall quality of life for both patients with cancer and their caregivers (43). Furthermore, comprehensive patient education remains instrumental in facilitating early diagnosis, enhancing treatment adherence, and optimizing clinical outcomes (44).

This study presents several limitations. Most significantly, the absence of CYP3A4/3A5 genotyping hindered stratification of patients by metabolizer status. Given that tacrolimus metabolism is pre-dominantly CYP3A-dependent, this constitutes a primary confounding factor. CYP3A5 expressers generally require doses up to 50% higher to reach target trough levels and exhibit greater pharmacokinetic variability. Consequently, an unequal distribution of expressers across the cohorts may introduce bias: a higher proportion of poor metabolizers in the intervention group could lead to an overestimation of the pharmacist's stabilizing influence, whereas an excess of rapid metabolizers might result in an underestimation.

Additionally, staff awareness of the study may have introduced a Hawthorne effect. We minimized this by evaluating objective pharmacokinetic biomarkers (trough levels) rather than subjective performance scales. Finally, the identification of patient-specific DRPs often relied on caregiver self-reporting, making the intervention vulnerable to recall bias.

Integrating a clinical pharmacist into the pediatric transplant care team significantly optimizes therapeutic outcomes. Through targeted education, proactive DRP resolution, and rigorous pharmacokinetic monitoring, the intervention corrected critical knowledge deficits, reduced medication-use risks, and accelerated time to therapeutic stability. Standardizing pharmacist-led therapeutic drug monitoring should be considered a necessary pillar in pediatric transplant units to ensure long-term graft survival.

## Data Availability

The original contributions presented in the study are included in the article/Supplementary material, further inquiries can be directed to the corresponding author.
