# Supplementary material for: Impact of clinical pharmacist-led medication management and education on tacrolimus therapeutic control
Source: Front Med (Lausanne). 2026 Jun 9;13:1851388. doi: 10.3389/fmed.2026.1851388 (PMC13286822; doi:10.3389/fmed.2026.1851388)
Supplement: Supplementary file 1 [file Table_1.docx]

Supplementary Table 1: **Informal caregiver: Tacrolimus knowledge scale assessment**

| **Item** | **Statement** | **Expected Answer** | **Pre-intervention (n=65)T: n (%)F: n (%)DK: n (%)** | **Immediately Post-intervention (n=64)*T: n (%)F: n (%)DK: n (%)** | **3-Month Follow-up (n=63)**T: n (%)F: n (%)DK: n (%)** | **p-value** |
| --- | --- | --- | --- | --- | --- | --- |
| **A1** | Tacrolimus is used to prevent organ rejection without causing severe harm to the body. | T | 42 (64.6)  0  23 (35.4) | 60 (93.8)  1 (1.6)  3 (4.7) | 63 (100.0)  0  0 | <0.001 |
| **A2** | Tacrolimus must be used for a long period following transplantation surgery. | T | 12 (18.5)  23 (35.4)  30 (46.2) | 64 (100.0)  0  0 | 48 (76.2)  11 (17.5)  4 (6.3) | <0.001 |
| **A3** | The patient can stop taking the medication when they feel well. | F | 7 (10.8)  47 (72.3)  11 (16.9) | 5 (7.8)  59 (92.2)  0 | 4 (6.3)  59 (93.8)  0 | <0.001 |
| **A4** | If the patient vomits within the first 2 hours after taking the medication, the same dose can be repeated; if vomiting occurs after 2 hours, the dose does not need to be repeated. | F | 14 (21.5)  24 (36.9)  27 (41.5) | 3 (4.7)  61 (95.3)  0 | 11 (17.5)  52 (82.5)  0 | <0.001 |
| **A5** | It is important to take tacrolimus at the same time every day. | T | 59 (90.8)  0  6 (9.2) | 59 (92.2)  5 (7.8)  0 | 60 (95.2)  3 (4.8)  0 | 0.002 |
| **A6** | The patient can take herbal supplements during this treatment process. | F | 4 (6.2)  44 (67.7)  17 (26.2) | 6 (9.4)  58 (90.6)  0 | 7 (11.1)  56 (90.5)  0 | <0.001 |
| **A7** | Tacrolimus prevents the rejection of the transplanted organ by weakening the white blood cells responsible for protecting the body. | T | 28 (43.1)  6 (9.2)  31 (47.7) | 59 (92.2)  5 (7.8)  0 | 57 (90.5)  6 (9.5)  0 | <0.001 |
| **A8** | If tacrolimus is used twice daily, it should be taken approximately every 12 hours. | T | 59 (90.8)  0  6 (9.2) | 63 (98.4)  1 (1.6)  0 | 62 (98.4)  1 (1.6)  0 | 0.010 |
| **A9** | Since tacrolimus can cause gastrointestinal issues, it is recommended to be taken before meals. | T | 27 (41.5)  21 (32.3)  17 (26.2) | 64 (100.0)  0  0 | 63 (100.0)  0  0 | <0.001 |
| **A10** | Grapefruit should not be eaten and grapefruit juice should not be consumed while taking tacrolimus. | T | 35 (53.8)  4 (6.2)  26 (40.0) | 64 (100.0)  0  0 | 62 (98.4)  0  1 (1.6) | <0.001 |
| **A11** | If the patient remembers a missed dose and there is less than 6 hours until the next dose, they can take the missed dose. | F | 18 (27.7)  11 (16.9)  36 (55.4) | 64 (100.0)  0  0 | 56 (88.9)  5 (7.9)  2 (3.2) | <0.001 |

Supplementary Table 2: **Informal caregiver**: Responses to the parental attitude scale for rational drug

| **Item** | **Statement** | **Pre-intervention (n=65)n (%)** | **Immediately Post-intervention (n=64)*n (%)** | **3-Month Follow-up (n=63)**n (%)** | **p-value** |
| --- | --- | --- | --- | --- | --- |
| **B1** | I receive information about my child's medications from healthcare professionals. | SA: 54 (83.08)  A: 9 (13.85)  U: 0  D: 2 (3.08)  SD: 0 | SA: 63 (98.44)  A: 0  U: 0  D: 0  SD: 1 (1.56) | SA: 59 (93.65)  A: 1 (1.59)  U: 0  D: 1 (1.59)  SD: 2 (3.17) | 0.003 |
| **B2** | I know what the medication I give my child is used for. | SA: 33 (50.77)  A: 22 (33.85)  U: 3 (4.62)  D: 0  SD: 7 (10.77) | SA: 64 (100.00)  A: 0  U: 0  D: 0  SD: 0 | SA: 56 (88.89)  A: 7 (11.11)  U: 0  D: 0  SD: 0 | <0.001 |
| **B3** | I check the prescription written for my child. | SA: 53 (81.54)  A: 12 (18.46)  U: 0  D: 0  SD: 0 | SA: 57 (89.06)  A: 7 (10.94)  U: 0  D: 0  SD: 0 | SA: 56 (88.89)  A: 7 (11.11)  U: 0  D: 0  SD: 0 | 0.330 |
| **B4** | I administer my child's medications exactly as prescribed. | SA: 60 (92.31)  A: 5 (7.69)  U: 0  D: 0  SD: 0 | SA: 64 (100.00)  A: 0  U: 0  D: 0  SD: 0 | SA: 63 (100.00)  A: 0  U: 0  D: 0  SD: 0 | 0.006 |
| **B5** | I prepare my child's suspension (powder for reconstitution) medications as instructed in the package insert. | SA: 50 (76.92)  A: 14 (21.54)  U: 1 (1.54)  D: 0  SD: 0 | SA: 64 (100.00)  A: 0  U: 0  D: 0  SD: 0 | SA: 63 (100.00)  A: 0  U: 0  D: 0  SD: 0 | <0.001 |
| **B6** | I know the side effects of the medications I give my child. | SA: 33 (50.77)  A: 11 (16.92)  U: 20 (30.77)  D: 1 (1.54)  SD: 0 | SA: 64 (100.00)  A: 0  U: 0  D: 0  SD: 0 | SA: 53 (84.13)  A: 10 (15.87)  U: 0  D: 0  SD: 0 | <0.001 |
| **B7** | I check the expiration date of the medications I give my child. | SA: 59 (90.77)  A: 6 (9.23)  U: 0  D: 0  SD: 0 | SA: 64 (100.00)  A: 0 U: 0  D: 0  SD: 0 | SA: 63 (100.00)  A: 0  U: 0 D: 0  SD: 0 | 0.002 |
| **B8** | I store my child's medications together with their package insert. | SA: 57 (87.69)  A: 7 (10.77)  U: 1 (1.54)  D: 0  SD: 0 | SA: 61 (95.31)  A: 3 (4.69)  U: 0  D: 0  SD: 0 | SA: 60 (95.24)  A: 3 (4.76)  U: 0  D: 0  SD: 0 | 0.315 |
| **B9** | I discard medications that I suspect have spoiled. | SA: 57 (87.69)  A: 5 (7.69)  U: 0  D: 0  SD: 3 (4.62) | SA: 64 (100.00)  A: 0  U: 0  D: 0  SD: 0 | SA: 63 (100.00) A: 0  U: 0  D: 0  SD: 0 | 0.002 |
| **B10** | I do not use medications recommended by others for my child. | SA: 50 (76.92)  A: 4 (6.15)  U: 0  D: 0  SD: 11 (16.92) | SA: 64 (100.00)  A: 0  U: 0  D: 0  SD: 0 | SA: 63 (100.00)  A: 0  U: 0  D: 0  SD: 0 | <0.001 |
| **B11** | I read the package insert of the medications I give my child. | SA: 59 (90.77)  A: 6 (9.23)  U: 0  D: 0  SD: 0 | SA: 64 (100.00)  A: 0  U: 0  D: 0  SD: 0 | SA: 63 (100.00)  A: 0  U: 0  D: 0  SD: 0 | 0.002 |
| **B12** | I store medications in places out of my child's reach. | SA: 59 (90.77)  A: 5 (7.69)  U: 1 (1.54)  D: 0  SD: 0 | SA: 64 (100.00)  A: 0  U: 0  D: 0  SD: 0 | SA: 63 (100.00)  A: 0  U: 0  D: 0  SD: 0 | 0.015 |
| **B13** | If a medication causes a side effect, I stop using it. | SA: 47 (72.31)  A: 7 (10.77)  U: 0  D: 2 (3.08)  SD: 9 (13.85) | SA: 64 (100.00)  A: 0  U: 0  D: 0  SD: 0 | SA: 63 (100.00)  A: 0 U: 0  D: 0  SD: 0 | <0.001 |
| **B14** | I do not give my child over-the-counter medications. | SA: 62 (95.38)  A: 3 (4.62)  U: 0  D: 0  SD: 0 | SA: 64 (100.00)  A: 0  U: 0  D: 0  SD: 0 | SA: 63 (100.00)  A: 0  U: 0  D: 0  SD: 0 | 0.048 |
| **B15** | I give my child their medication for the recommended duration. | SA: 61 (93.85)  A: 4 (6.15)  U: 0  D: 0  SD: 0 | SA: 64 (100.00)  A: 0  U: 0  D: 0  SD: 0 | SA: 63 (100.00)  A: 0  U: 0  D: 0  SD: 0 | 0.017 |
| **B16** | If I need to give my child multiple medications, I mix them together. *(negative item)* | SA: 61 (93.85)  A: 4 (6.15) U: 0  D: 0  SD: 0 | SA: 6 (9.38)  A: 7 (10.94)  U: 0  D: 12 (18.75)  SD: 39 (60.94) | SA: 8 (12.70)  A: 7 (11.11)  U: 0  D: 12 (19.05)  SD: 36 (57.14) | <0.001 |
| **B17** | I shake suspensions (powders reconstituted with water) before administering them to my child. | SA: 13 (20.00)  A: 4 (6.15)  U: 0  D: 14 (21.54)  SD: 34 (52.31) | SA: 62 (96.88)  A: 1 (1.56)  U: 0  D: 1 (1.56)  SD: 0 | SA: 61 (96.83)  A: 1 (1.59)  U: 0  D: 1 (1.59)  SD: 0 | <0.001 |
| **B18** | When storing my child's medication, I pay attention to the storage conditions written in the package insert. | SA: 61 (93.80)  A: 2 (3.08)  U: 0  D: 2 (3.08)  SD: 0 | SA: 64 (100.00)  A: 0  U: 0  D: 0  SD: 0 | SA: 63 (100.00)  A: 0  U: 0  D: 0  SD: 0 | 0.086 |
| **B19** | I store my child's medication in its original box to protect it from light. | SA: 62 (95.38)  A: 1 (1.54)  U: 0  D: 2 (3.08)  SD: 0 | SA: 64 (100.00)  A: 0  U: 0  D: 0  SD: 0 | SA: 63 (100.00)  A: 0  U: 0  D: 0  SD: 0 | 0.192 |
| **B20** | I give my child their medication at the recommended time intervals. | SA: 62 (95.38)  A: 1 (1.54)  U: 0  D: 2 (3.08)  SD: 0 | SA: 64 (100.00)  A: 0  U: 0  D: 0  SD: 0 | SA: 63 (100.00)  A: 0  U: 0  D: 0  SD: 0 | 0.192 |
| **B21** | Unnecessary medication use is harmful to health. | SA: 60 (92.31)  A: 2 (3.08)  U: 1 (1.54)  D: 2 (3.08)  SD: 0 | SA: 64 (100.00)  A: 0  U: 0  D: 0  SD: 0 | SA: 63 (100.00)  A: 0  U: 0  D: 0  SD: 0 | 0.114 |
| **B22** | If I do not see a benefit from the medication I use for my child, I consult a doctor. | SA: 56 (86.15)  A: 4 (6.15)  U: 2 (3.08)  D: 3 (4.62)  SD: 0 | SA: 64 (100.00)  A: 0  U: 0  D: 0  SD: 0 | SA: 63 (100.00)  A: 0  U: 0  D: 0  SD: 0 | 0.004 |
| **B23** | Before giving my child their medication, I check whether it should be taken on an empty or full stomach. | SA: 57 (97.69)  A: 6 (9.23)  U: 0  D: 2 (3.08)  SD: 0 | SA: 64 (100.00)  A: 0  U: 0  D: 0  SD: 0 | SA: 63 (100.00)  A: 0  U: 0  D: 0  SD: 0 | 0.002 |
| **B24** | I give my child their medication using the measuring spoon provided in the box. | SA: 60 (92.31)  A: 3 (4.62)  U: 0  D: 2 (3.08)  SD: 0 | SA: 62 (96.88)  A: 2 (3.13)  U: 0  D: 0  SD: 0 | SA: 63 (100.00)  A: 0  U: 0  D: 0  SD: 0 | 0.163 |
| **B25** | I give my child their medication in the recommended dose/amount. | SA: 61 (93.85)  A: 2 (3.08)  U: 0  D: 2 (3.08)  SD: 0 | SA: 64 (100.00)  A: 0  U: 0  D: 0  SD: 0 | SA: 63 (100.00)  A: 0  U: 0  D: 0  SD: 0 | 0.086 |
| **B26** | If my child's suspension (powder to be reconstituted with water) medication is not finished within 10 days, I discard the remaining amount. | SA: 51 (78.46)  A: 1 (1.54)  U: 3 (4.62)  D: 2 (3.08)  SD: 8 (12.31) | SA: 64 (100.00)  A: 0  U: 0  D: 0  SD: 0 | SA: 63 (100.00)  A: 0  U: 0  D: 0  SD: 0 | <0.001 |
| **B27** | I use boiled and cooled water when preparing my child's suspension (powder to be reconstituted with water) medication. | SA: 60 (92.31)  A: 3 (4.62)  U: 0  D: 2 (3.08)  SD: 0 | SA: 64 (100.00)  A: 0 (0.00)  U: 0  D: 0  SD: 0 | SA: 63 (100.00)  A: 0  U: 0  D: 0  SD: 0 | 0.036 |
| **B28** | If a side effect occurs in my child due to the medication I gave, I consult a doctor. | SA: 62 (95.38)  A: 1 (1.54)  U: 0  D: 2 (3.08)  SD: 0 | SA: 64 (100.00)  A: 0  U: 0  D: 0  SD: 0 | SA: 63 (100.00)  A: 0  U: 0  D: 0  SD: 0 | 0.192 |
| **B29** | I do not give my child non-prescription antibiotics. | SA: 62 (95.38)  A: 1 (1.54)  U: 0  D: 2 (3.08)  SD: 0 | SA: 64 (100.00)  A: 0  U: 0  D: 0  SD: 0 | SA: 63 (100.00)  A: 0  U: 0  D: 0  SD: 0 | 0.192 |
| **B30** | When my child gets sick, I give them medications available at home before going to a healthcare facility. *(negative item)* | SA: 12 (18.46)  A: 6 (9.23)  U: 6 (9.23)  D: 1 (1.54) SD: 40 (61.54) | SA: 21 (32.81)  A: 7 (10.94)  U: 0  D: 0  SD: 36 (56.25) | SA: 21 (33.33)  A: 0  U: 7 (11.11)  D: 2 (3.17)  SD: 33 (52.38) | 0.017 |
| **B31** | When my child gets sick, I alternate between multiple medications that have the same effect. *(negative item)* | SA: 34 (52.31)  A: 3 (4.62)  U: 2 (3.08)  D: 0  SD: 26 (40.00) | SA: 48 (75.00)  A: 0  U: 0  D: 0  SD: 16 (25.00) | SA: 47 (74.60)  A: 0  U: 0  D: 0  SD: 16 (25.40) | 0.010 |
| **B32** | I give my child their capsule medication by opening the capsule. *(negative item)* | SA: 13 (20.00)  A: 3 (4.62)  U: 2 (3.08)  D: 4 (6.15)  SD: 43 (66.15) | SA: 3 (4.69)  A: 25 (39.06)  U: 3 (4.69)  D: 0  SD: 33 (51.56) | SA: 3 (4.76)  A: 24 (38.10)  U: 4 (6.35)  D: 0  SD: 32 (50.79) | <0.001 |
| **B33** | I recommend a medication that I think is effective for my child to others. *(negative item)* | SA: 1 (1.54)  A: 1 (1.54)  U: 1 (1.54)  D: 4 (6.15)  SD: 58 (89.23) | SA: 2 (3.13)  A: 0  U: 0  D: 0  SD: 62 (96.88) | SA: 2 (3.17)  A: 0  U: 0  D: 0  SD: 61 (96.83) | 0.124 |
| **B34** | If a side effect occurs in my child due to the medication I gave, I try to find a solution on my own. *(negative item)* | SA: 2 (3.08)  A: 1 (1.54)  U: 5 (7.69)  D: 5 (7.69)  SD: 52 (80.00) | SA: 2 (3.13)  A: 0  U: 0  D: 0  SD: 62 (96.88) | SA: 2 (3.17)  A: 0  U: 0  D: 0  SD: 61 (96.83) | 0.003 |
| **B35** | I get information about my child's medication from people around me. *(negative item)* | SA: 7 (10.77)  A: 5 (7.69)  U: 0  D: 2 (3.08)  SD: 51 (78.46) | SA: 2 (3.13)  A: 0  U: 0  D: 0  SD: 62 (96.88) | SA: 2 (3.17)  A: 0  U: 0  D: 0  SD: 61 (96.83) | 0.003 |
| **B36** | I give my child their medication with milk. *(negative item)* | SA: 17 (26.15)  A: 4 (6.15)  U: 5 (7.69)  D: 2 (3.08)  SD: 37 (56.92) | SA: 2 (3.13)  A: 0  U: 7 (10.94)  D: 0  SD: 55 (85.94) | SA: 2 (3.17)  A: 7 (11.11)  U: 0  D: 0  SD: 54 (85.71) | <0.001 |
| **B37** | I crush my child's tablets before giving them. *(negative item)* | SA: 9 (13.85)  A: 4 (6.15)  U: 1 (1.54)  D: 4 (6.15)  SD: 47 (72.31) | SA: 9 (14.06)  A: 0  U: 15 (23.44)  D: 1 (1.56)  SD: 40 (60.94) | SA: 9 (14.29)  A: 0  U: 15 (23.81)  D: 1 (1.59)  SD: 38 (60.32) | 0.002 |
| **B38** | When my child shows symptoms similar to previous ones, I give them medications from home without consulting anyone. *(negative item)* | SA: 2 (3.08)  A: 6 (9.23) U: 0  D: 11 (16.92)  SD: 46 (70.77) | SA: 2 (3.13)  A: 0  U: 0  D: 7 (10.94)  SD: 55 (85.94) | SA: 2 (3.17)  A: 0 U: 0  D: 7 (11.11)  SD: 54 (85.71) | 0.024 |
| **B39** | After my child's medication treatment is finished, I keep the leftover medication at home. *(negative item)* | SA: 5 (7.69)  A: 8 (12.31)  U: 3 (4.62)  D: 11 (16.92)  SD: 38 (58.46) | SA: 2 (3.13)  A: 0  U: 0  D: 23 (35.94)  SD: 40 (60.94) | SA: 2 (3.17)  A: 11 (17.46)  U: 0  D: 12 (19.05)  SD: 38 (60.32) | 0.002 |
| **B40** | I give my child their medication with fruit juice. *(negative item)* | SA: 7 (10.77)  A: 10 (15.38)  U: 2 (3.08)  D: 2 (3.08)  SD: 44 (67.69) | SA: 2 (3.13)  A: 7 (10.94)  U: 0 (0.00)  D: 17 (26.56)  SD: 39 (59.38) | SA: 2 (3.17)  A: 1 (1.59)  U: 6 (9.52)  D: 17 (26.98)  SD: 37 (58.73) | <0.001 |

**SA** = Strongly Agree; **A** = Agree; **U** = Undecided/Neutral; **D** = Disagree; **SD** = Strongly Disagree; **n** = number of participants. ** Data loss occurred due to the death of one patient prior to the immediate post-intervention test.* *An additional patient died during the 1-to-3-month follow-up period, bringing the total number of deceased patients to two.*

Supplementary Table 3: **Nursing outcomes: Tacrolimus knowledge scale assessment (N=50)**

| **Item** | **Statement** | **Expected Answer** | **Pre-interventionT: n (%)F: n (%)DK: n (%)** | **Immediately Post-interventionT: n (%)F: n (%)DK: n (%)** | **3-Month Follow-upT: n (%)F: n (%)DK: n (%)** | **p-value** |
| --- | --- | --- | --- | --- | --- | --- |
| **X1** | Tacrolimus is used to prevent organ rejection without causing severe harm to the body. | T | 35 (70.0)  7 (14.0)  8 (16.0) | 50 (100.0)  0  0 | 50 (100.0)  0  0 | <0.001 |
| **X2** | Tacrolimus must be used for a long period following transplantation surgery. | T | 19 (38.0)  20 (40.0)  11 (22.0) | 50 (100.0)  0  0 | 35 (70.0)  12 (24.0)  3 (6.0) | <0.001 |
| **X3** | The patient can stop taking the medication when they feel well. | F | 2 (4.0)  48 (96.0)  0 | 0  50 (100.0)  0 | 0  50 (100.0)  0 | 0.132 |
| **X4** | If the patient vomits within the first 2 hours after taking the medication, the same dose can be repeated; if vomiting occurs after 2 hours, the dose does not need to be repeated. | F | 28 (56.0)  20 (40.0)  2 (4.0) | 3 (6.0)  47 (94.0)  0 | 12 (24.0)  38 (76.0)  0 | <0.001 |
| **X5** | It is important to take tacrolimus at the same time every day. | T | 44 (88.0)  4 (8.0)  2 (4.0) | 50 (100.0)  0  0 | 50 (100.0)  0  0 | 0.014 |
| **X6** | The patient can take herbal supplements during this treatment process. | F | 11 (22.0)  32 (64.0)  7 (14.0) | 0  50 (100.0)  0 | 0  50 (100.0)  0 | <0.001 |
| **X7** | Tacrolimus prevents the rejection of the transplanted organ by weakening the white blood cells responsible for protecting the body. | T | 42 (84.0)  6 (12.0)  2 (4.0) | 50 (100.0)  0  0 | 50 (100.0)  0  0 | 0.002 |
| **X8** | If tacrolimus is used twice daily, it should be taken approximately every 12 hours. | T | 48 (96.0)  0  2 (4.0) | 50 (100.0)  0  0 | 50 (100.0)  0  0 | 0.132 |
| **X9** | Since tacrolimus can cause gastrointestinal issues, it is recommended to be taken before meals. | T | 17 (34.0)  13 (26.0)  20 (40.0) | 50 (100.0)  0  0 | 50 (100.0)  0  0 | <0.001 |
| **X10** | Grapefruit should not be eaten and grapefruit juice should not be consumed while taking tacrolimus. | T | 35 (70.0)  5 (10.0)  10 (20.0) | 50 (100.0)  0  0 | 50 (100.0)  0  0 | <0.001 |
| **X11** | If the patient remembers a missed dose and there is less than 6 hours until the next dose, they can take the missed dose. | F | 12 (24.0)  24 (48.0)  14 (28.0) | 50 (100.0)  0  0 | 48 (96.0)  2 (4.0)  0 | <0.001 |

**T** = True; **F** = False; **DK** = Don't Know; **n** = number of participants.

Supplementary Table 4: **Responses to the food-drug interaction knowledge scale for healthcare professionals**

| **Item** | **Statement** | **Expected Answer** | **Pre-interventionT: n (%)F: n (%)DK: n (%)** | **Immediately Post-interventionT: n (%)F: n (%)DK: n (%)** | **3-Month Follow-upT: n (%)F: n (%)DK: n (%)** | **p-value** |
| --- | --- | --- | --- | --- | --- | --- |
| **Y1** | A person's fasting state does not alter the efficacy of a drug. | F | 21 (31.0)  29 (69.0)  0 | 0  50 (100.00)  0 | 0  50 (100.00  0 | <0.001 |
| **Y2** | Some antiulcer drugs, such as sucralfate, should be taken on an empty stomach because they bind to proteins in food. | T | 39 (78.0)  4 (8.0)  7 (14.0) | 50 (100.00)  0  0 | 50 (100.00)  0  0 | <0.001 |
| **Y3** | Energy-restricted diets can increase sensitivity to stimulant drugs such as amphetamines. | T | 33 (66.0)  2 (4.0)  15 (30.0) | 29 (58.00) 21 (42.00)  0 | 29 (58.00)  11 (22.00)  10 (20.00) | <0.001 |
| **Y4** | Taking a lipophilic drug with a high-fat meal increases drug toxicity. | T | 29 (58.0)  12 (24.0)  9 (18.0) | 50 (100.00)  0  0 | 50 (100.00)  0  0 | <0.001 |
| **Y5** | A high-carbohydrate diet decreases the excretion of certain anti-asthma drugs, such as theophylline. | F | 35 (70.00)  0  15 (30.00) | 0  50 (100.00)  0 | 0  30 (60.00)  20 (40.00) | <0.001 |
| **Y6** | The bioavailability of propranolol increases when taken with a high-carbohydrate meal. | F | 14 (28.00)  7 (14.00)  29 (58.00) | 0  50 (100.00) 0 | 0  50 (100.00)  0 | <0.001 |
| **Y7** | High-fiber diets increase the excretion of certain drugs by binding to bile acids. | T | 30 (60.0)  6 (12.0)  14 (28.0) | 50 (100.00)  0  0 | 50 (100.00)  0  0 | <0.001 |
| **Y8** | Foods high in fiber and pectin delay the absorption of certain drugs, such as digoxin. | T | 29 (58.00)  2 (4.00)  19 (38.00) | 50 (100.00)  0  0 | 29 (58.00)  7 (14.00)  14 (28.00) | <0.001 |
| **Y9** | A low-protein diet reduces drug half-life and plasma clearance by lowering albumin levels. | F | 35 (70.00)  2 (4.00)  13 (26.00) | 29 (58.00)  21 (42.00)  0 | 20 (40.00)  30 (60.00)  0 | <0.001 |
| **Y10** | Grapefruit juice increases the blood concentration of calcium channel blockers, such as felodipine. | T | 22 (44.00)  14 (28.00)  14 (28.00) | 34 (68.00)  16 (32.00)  0 | 28 (56.00)  16 (32.00)  6 (12.00) | 0.002 |
| **Y11** | The bioavailability of chemotherapeutic drugs increases with grapefruit juice. | T | 22 (44.00)  12 (24.00)  16 (32.00) | 16 (32.00)  34 (68.00)  0 | 25 (50.00)  15 (30.00)  10 (20.00) | <0.001 |
| **Y12** | Consuming caffeinated beverages with bisphosphonates increases drug absorption and efficacy. | F | 20 (40.00)  5 (10.00)  25 (50.00) | 34 (68.00)  16 (32.00)  0 | 21 (42.00)  26 (52.00)  3 (6.00) | <0.001 |
| **Y13** | Vegetables rich in vitamin K increase the efficacy of anticoagulants containing warfarin. | F | 36 (72.00)  2 (4.00)  12 (24.00) | 16 (32.00)  34 (68.00)  0 | 11 (22.00)  31 (62.00)  8 (16.00) | <0.001 |
| **Y14** | Taking levodopa or methyldopa with protein-rich foods decreases drug absorption. | T | 12 (24.00)  5 (10.00)  33 (66.00) | 37 (74.00)  13 (26.00)  0 | 23 (46.00)  13 (26.00)  14 (28.00) | <0.001 |
| **Y15** | Antibiotics such as tetracycline and ciprofloxacin bind to calcium, magnesium, iron, and zinc, increasing the absorption of both the drug and the mineral. | F | 17 (34.00)  0  33 (66.00) | 16 (32.00)  34 (68.00)  0 | 13 (26.00)  34 (68.00)  3 (6.00) | <0.001 |
| **Y16** | Anticonvulsants such as phenobarbital and phenytoin can disrupt the metabolism of folic acid, vitamin D, and vitamin K. | T | 19 (38.00)  6 (12.00)  25 (50.00) | 34 (68.00)  16 (32.00)  0 | 24 (48.00)  13 (26.00)  13 (26.00) | <0.001 |
| **Y17** | Antacids such as sodium bicarbonate decrease calcium absorption. | T | 22 (44.00)  10 (20.00)  18 (36.00) | 13 (26.00)  37 (74.00)  0 | 13 (26.00)  29 (58.00)  8 (16.00) | <0.001 |
| **Y18** | Antihypertensives containing ACE inhibitors should not be consumed with potassium-rich foods due to the risk of hyperkalemia. | T | 9 (18.00)  12 (24.00)  29 (58.00) | 50 (100.00)  0  0 | 32 (64.00)  0  18 (36.00) | <0.001 |
| **Y19** | Long-term steroid use negatively affects calcium metabolism. | T | 29 (58.00)  0  21 (42.00) | 13 (26.00)  37 (74.00)  0 | 20 (40.00)  21 (42.00)  9 (18.00) | <0.001 |
| **Y20** | Immunosuppressive drugs containing cyclosporine can increase blood pressure by enhancing sodium and water retention. | T | 33 (66.00)  0  17 (34.00) | 50 (100.00)  0 0 | 30 (60.00)  6 (12.00)  14 (28.00) | <0.001 |
| **Y21** | Thiazide and loop diuretics trigger hypokalemia by increasing potassium excretion. | T | 10 (20.00)  6 (12.00)  34 (68.00) | 13 (26.00)  37 (74.00)  0 | 24 (48.00)  18 (36.00)  8 (16.00) | <0.001 |
| **Y22** | Antacids and proton pump inhibitors negatively affect iron absorption by altering gastric pH. | T | 16 (32.00)  6 (12.00)  28 (56.00) | 50 (100.00)  0  0 | 31 (62.00)  11 (22.00)  8 (16.00) | <0.001 |
| **Y23** | MAO inhibitors can cause a hypertensive crisis when taken with tyramine-rich foods. | T | 10 (20.00)  0  40 (80.00) | 29 (58.00)  21 (42.00)  0 | 29 (58.00)  21 (42.00)  0 | <0.001 |
| **Y24** | Statin-group antihyperlipidemic drugs increase the absorption of fat-soluble vitamins. | F | 16 (32.00)  0  34 (68.00) | 21 (42.00)  29 (58.00)  0 | 16 (32.00)  29 (58.00)  5 (10.00) | <0.001 |
| **Y25** | Antidiabetic drugs containing metformin negatively affect vitamin B12 absorption. | T | 28 (56.00)  0  22 (44.00) | 50 (100.00)  0  0 | 28 (56.00)  9 (18.00)  13 (26.00) | <0.001 |

**T** = True; **F** = False; **DK** = Don't Know; **n** = number of participants.
